# Oregonin from *Alnus incana* bark affects DNA methyltransferases expression and mitochondrial DNA copies in mouse embryonic fibroblasts 

**DOI:** 10.1080/14756366.2018.1476504

**Published:** 2018-06-07

**Authors:** Jelena Krasilnikova, Liga Lauberte, Elena Stoyanova, Desislava Abadjieva, Mihail Chervenkov, Mattia Mori, Elisa De Paolis, Vanya Mladenova, Galina Telysheva, Bruno Botta, Elena Kistanova

**Affiliations:** a Department of Biochemistry, Stradiņš University, Riga, Latvia;; b Latvian State Institute of Wood Chemistry, Riga, Latvia;; c Institute of Biology and Immunology of Reproduction, Bulgarian Academy of Sciences, Sofia, Bulgaria;; d Faculty of Veterinary Medicine, University of Forestry, Sofia, Bulgaria;; e Institute of Neurobiology, Bulgarian Academy of Sciences, Sofia, Bulgaria;; f Center for Life Nano Science@Sapienza, Istituto Italiano di Tecnologia, Rome, Italy;; g Department of Chemistry and Technology of Drugs, Sapienza University of Rome, Rome, Italy

**Keywords:** Oregonin, *Alnus incana* bark, DNA methyltransferases mRNAs, mtDNA copy

## Abstract

Oregonin is an open-chain diarylheptanoid isolated from *Alnus incana* bark that possesses remarkable antioxidant and anti-inflammatory properties, inhibits adipogenesis, and can be used in the prevention of obesity and related metabolic disorders. Here, we aimed to investigate the effects of oregonin on the epigenetic regulation in cells as well as its ability to modulate DNA methylating enzymes expression and mitochondrial DNA (mtDNA) copies. Our results show that oregonin altered the expression of DNA methyltransferases and mtDNA copy numbers in dependency on concentration and specificity of cells genotype. A close correlation between mtDNA copy numbers and mRNA expression of the *mtDnmt1* and *Dnmt3b* was established. Moreover, molecular modeling suggested that oregonin fits the catalytic site of DNMT1 and partially overlaps with binding of the cofactor. These findings further extend the knowledge on oregonin, and elucidate for the first time its potential to affect the key players of the DNA methylation process, namely DNMTs transcripts and mtDNA.

## 1. Introduction

**Figure 1.**
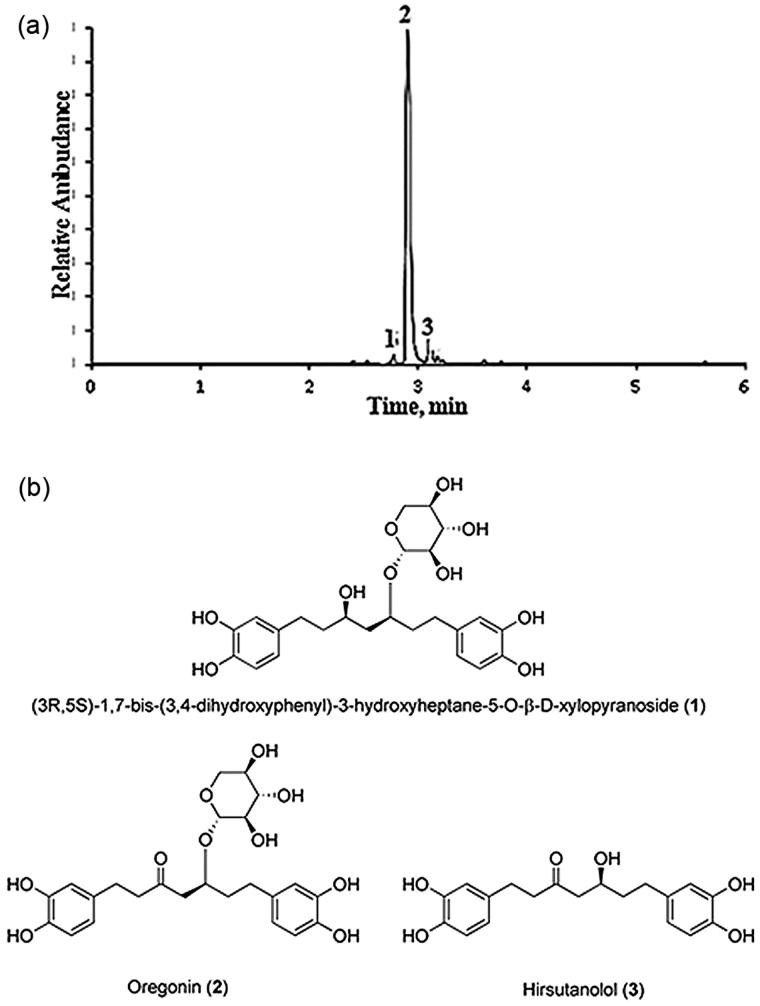
(a) Base peak ion chromatogram of purified (90% oregonin) alder bark extracts obtained by negative ion UPLC-ESI-MS. Oregonin (**2**), (3R,5S)-1,7-bis-(3,4-dihydroxyphenyl)-3-hydroxyheptane-5-O-β-d-xylopyranoside (**1**) and hirsutanolol (**3**). (b) Chemical structure of compounds **1–3**.

Oregonin 1,7-bis-(3,4-dihydroxyphenyl)-3- hydroxyheptane-5-O-b-D-xylopyranoside (Figure 1) is an open-chain diarylheptanoid glycoside that contains 3-carbonyl and 5-xylosyloxy groups, and is the predominant compound in the hydrophilic extracts from the *Alnus incana* bark^1^^,^^2^. This natural compound possesses noticeable anti-oxidative and anti-inflammatory properties, inhibits the adipogenesis and can be used in the prevention of obesity and related metabolic disorders^3–8^.

The effects of oregonin, isolated from the bark of grey alders growing in Latvia, have been tested *in vitro* on blood samples from volunteers with clinically confirmed metabolic syndromes. The total and low-density cholesterol as well as the level of triacylglycerols is reduced by 30%, 20% and 30%, respectively, after incubation with alder bark ethyl acetate extract containing 60% of oregonin or with purified oregonin for 30 min^1^. The effect has also been confirmed in white rats *in vivo*^1^^,^^9^. Pure oregonin from *A. incana* increases glutathione peroxidase and superoxide dismutase activity, and decreases the activity of lipase, catalase and malondialdehyde level (indicator of lipid peroxidation) in blood *in vivo*^1^^,^^9^. Also, it reduces lipid accumulation, inflammation and ROS production in primary human macrophages, indicating its anti-inflammatory activity^10^.

Oregonin shares chemical features and substructure with the well-known dietary antioxidant curcumin, which is widely used as a bioactive constituent of food supplements. Some studies^9^^,^^11^ have shown that oregonin expressed higher antioxidant activity and some others biological activities, particularly antibacterial and antifungal, than curcumin. While curcumin potential role in the epigenetic changes is described^12^^,^^13^, to the best of our knowledge no reports on the effects of oregonin from *A. incana* on the epigenetic regulation in cells are available to date.

Epigenetic changes cover altered DNA methylation, histone modification, non-coding RNAs and chromatin remodeling^14^^,^^15^. DNA methylation is regulated by enzymes belonging to the DNA methyltransferases (DNMTs) family, which transfer methyl groups from S-adenosyl-l-methionine (SAM) to the 5-position of the cytosine pyrimidine ring^16^. Hyper-methylation of DNA often occurs in gene-specific promoters mostly in gene-rich genomic regions (CpG-islands) and can lead to the inhibition of gene expression (gene silencing), while hypo-methylation is observed in repetitive elements all over the genome^14^^,^^17^. Increasing evidences are accumulating on the importance of mitochondria in the epigenetic regulation of cells. It is known that mitochondria are able to influence cytosine methylation levels in the nucleus by modulating the flux of one-carbon units for the generation of SAM, the methyl donor in DNA methylation^18^. As reported by Smiraglia et al.^19^, many genes undergo changes in their methylation status in response to the depletion and repletion of mtDNA. Mitochondrial dysfunction could cause alterations in redox (reduced oxygen) reactions and SAM-CH_3_ production in the cell, leading to perturbed methylation of nuclear encoded genomic DNA^18^.

A strong correlation between epigenetic alterations, including mitochondrial factors, and human diseases, especially cancer, has been observed^19–21^. Experimental evidences have pointed that polyphenols have great potential as epigenetic modulators, influencing various epigenetic factors such as histone acetyltransferases^22^, DNTMs and miRNAs^23–26^. These findings^13^^,^^27–29^ have increased the enthusiasm in developing therapeutic strategies for many diseases through targeting the various epigenetic factors by polyphenols. Accordingly, the identification of new natural and nontoxic polyphenols, able to affect reversibly the epigenetic deregulation, is nowadays a well-established and promising scientific topic.

Using a combination of biological and computational modeling tools, the present study aims to investigate whether the isolated and purified polyphenol oregonin from *A. incana* bark can affect the key players of the DNA methylation process, namely DNA methylating enzymes and mitochondrial DNA (mtDNA) in mouse embryonic fibroblasts (MEFs).

## 2. Materials and methods

### 2.1. Oregonin extraction

Bark of *A. incana* (Grey alder) was collected in November 2015 from trees growing in Latvia. Diarylheptanoids were obtained using accelerated solvent extraction with ethyl acetate after removal of lipophilic extractives (EtOAc). The bark was extracted with pressurised ethyl acetate in an ASE 350 apparatus (Dionex, Sunnyvale, CA) by applying 3 × 5 min static cycles at 90 °C and 10.3 MPa. The solvent was evaporated *in vacuo*, the extract was freeze-dried and stored at −20 °C.

Analysis of *A. incana* extract was performed on ACQUITY UPLC H-Class (Waters, Singapore) with ACQUITY UPLC PDA Detector coupled with SYNAPT G2-Si High Definition Mass Spectrometry (Waters, Milford, Massachusetts, MA, USA). Column was Waters ACQUITY UPLC BEH C18, 2.1 × 50 mm, 1.7 µm, flow 0.5 ml/min, Mobile phase A: waters +0.1% formic acid, B: acetonitrile, gradient from 10% A to 100% B, injection 2 µL. Mass range (50–1200 Da), ESI negative mode, cone voltage 40 V, scan time 0.1 s, desolvation gas nitrogen (600 L/h), source temperature at 120 °C.

Oregonin was purified from alder bark EtOAc extract by SP1™ Purification System using Biotage column KP-C18-HS (12 × 150 mm, 35–70 μm) with solvent systems: solvent A = ethanol/water/acetic acid (200: 790: 10, v/v) and solvent B = ethanol. The gradient was from 0 to 25% solvent B. Identification and purity state of oregonin was determined using UPLC-PDA-MS/MS, NMR methods. The proof quality is assessed by comparison to the oregonin reference standard [≥95% (LC/MS-ELSD)] purchased from Sigma-Aldrich (Saint Louis, MA, USA). For the investigation of antioxidant activity, the methods of the deactivation of 2,2-diphenyl-1-picrylhydrazyl (DPPH^•^) and 2,2′-azinobis-(3-ethylbenzothiazoline-6-sulfonic acid (ABTS^•+^) radicals were applied.

### 2.2. Animals

The animals used for obtaining the mouse embryonic cells were reared and handled in vivarium of the IBIR-BAS in accordance with Bulgarian Veterinary Law (25/01/2011) regarding the life conditions and welfare of experimental animals, which is adapted to the European Union regulation 86/609 and Directive 2010/63/EU. The experimental protocols were approved by the National Ethics commission for animals (“Permission for using animals in the experiments”, N84/04.10.2013, expiry date 04.10.2018). The individual responsible for the animal welfare, which works directly with the animals used in the study, is licensed by the Bulgarian veterinary services for animal experimentation. Animals were anesthetised before sacrificing them. All efforts were made to minimise suffering.

### 2.3. Cell cultures

MEFs were prepared from 13- to 14-day-old embryos of ICR mice. After dissection of the red organs, the tissues were minced and digested with 0.25% trypsin/EDTA (Sigma-Aldrich, Saint Louis, MA, USA). Dissociated cells were grown in monolayers at 37 °C in a humidified atmosphere of 5% CO_2_ and 95% air using Dulbecco’s Modified Eagle Medium (DMEM) with 1.0 g/L glucose (PAN-Biotech, Aidenbach, Germany) as the culture medium. DMEM was supplemented with 10% fetal bovine serum (Sigma-Aldrich , Saint Louis, MA, USA) and penicillin, streptomycin, amphotericin B mix (PAN-Biotech, Aidenbach, Germany). Mouse embryo fibroblast cell line, NIH/3T3, was obtained from the American-Type Culture Collection (Manassas, VA, USA). Cells were cultured under the conditions used for MEFs.

### 2.4. Cell viability assay

Cell viability was estimated by 3-(4,5-dimethylthiazol-2-yl)-2,5-diphenyltetrazolium bromide (MTT) assay (Sigma-Aldrich, Saint Louis, MA, USA), which is based on the cleavage of a tetrazolium salt by mitochondrial dehydrogenases in viable cells. NIH/3T3 cells and MEFs were seeded in sterile flat bottom 96-well plates at a density of approximately 2 × 10^4^ cells/well and allowed to attach overnight at 37 °C. The cells were then exposed to 50 and 100 μM oregonin dissolved in culture medium for 24 and 48 h. Untreated cells and blank wells (without cells) received 100 μl of the culture medium. After incubation with oregonin, the culture medium was removed and 100 μl of MTT at the concentration of 500 μg/ml in culture medium was added to the wells. Cells were incubated in a humidified CO_2_ incubator for another 3 h. Then, the MTT solution was removed and 100 μl of dimethyl sulfoxide (DMSO, Merck, Darmstadt, Germany) was added to dissolve formazan crystals. Absorbance at 544 nm was monitored with a plate reader (FLUOstar Optima, BMG Labtech, Offenburg, Germany) to obtain the number of viable cells relative to the control population. Data are expressed as mean values ± SD and obtained from three different experiments against each cell type.

### 2.5. Quantitative real-time PCR (qRT-PCR) assay

Fibroblasts were plated in sterile six-well plates at a density 2 × 10^5^ cells/well and were left to adhere overnight. Cells were treated with 50 and 100 μM oregonin in culture medium for 24 h. The samples were collected and subjected by TriReagent (Sigma-Aldrich, Saint Louis, MA, USA) in accordance with producers’ instruction for total RNA and genomic DNA extractions.

#### 2.5.1. Dnmts mRNA expression

The extracted RNA was used for the synthesis of the first cDNA strands using cDNA synthesis kit (Thermo Fisher Scientific, Waltham, CA, USA). qRT-PCR was performed on Dnmt1; *mtDnmt1*, *Dnmt3a* and *Dnmt3b* genes using SYBR Green PCR Master Mix (Life Technologies, Waltham, CA, USA). The primers are presented in Table 1. β*-actin* was selected as a housekeeping gene.

**Table 1.** Primer sequences for mouse *Dnmt* genes and β*-actin.*

Genes	Sequence (5′→3′)	Size (bp)	*T*_m_^b^ (°C)
*Dnmt1*	F: GGGTCTCGTTCAGAGCTG	201	60
	R: GCAGGAATTCATGCAGTAAG		
*mtDnmt1*	F: TCTCTTGCCCTGTGTGGTACATG	164	60
	R: TCTTTCCAAGTCTTTGAGCCGCC		
*Dnmt3a*	F: CCGCCTCTTCTTTGAGTTCTAC	125	55
	R: AGATGTCCCTCTTGTCACTAACG		
*Dnmt3b*	F: ATGGAGATCAGGAGGGTATGGA	177	56
	R: GTCGCTTGGAGGTGGCTTTC		
β*-actin*	F: GACCCAGATCATGTTTGAGACC	122	60
	R: ATCAGAATGCCTGTGGTACGAC		

**Figure 2.**
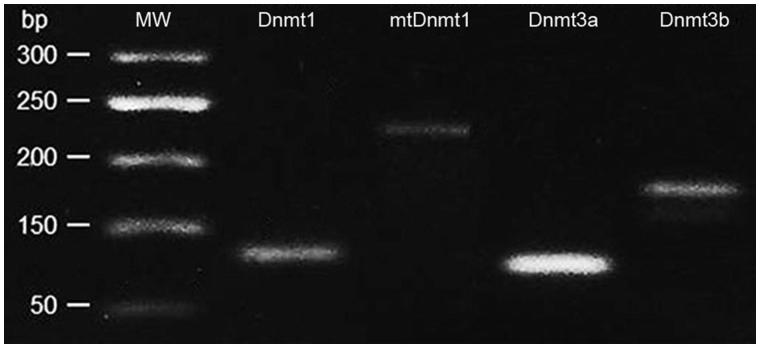
Agarose gel electrophoresis of PCR product (*Dnmts*).

All primers were obtained from Sigma-Aldrich, Saint Louis, MA, USA. PCR analysis was performed using a two-step cycling protocol, which was optimised under the following conditions: cycles consisted of a 10-min initial denaturation phase at 95 °C, a 15-s denaturation phase at 95 °C and a 20-s annealing phase based on the Tm of the primers (55–60 °C) with the last two steps repeated for a total of 40 cycles. A melting curve was made after cycling by changing the temperature from 60 to 90 °C and read and recorded every 1 °C. The process was implemented on Mastercycler^®^ ep realplex (Eppendorf, Hamburg, Germany). The presence of all *Dnmts* forms in the PCR product was confirmed by gel electrophoresis (Figure 2).

#### 2.5.2. mtDNA copy number evaluation

The purity and concentration of extracted DNA was measured using Nano Drop 1000 (Thermo Fisher Scientific, Waltham, MA USA). A ratiometric assay^30^ of the levels of a single mitochondrial gene cytochrome B (*Cytb*) against a single copy nuclear gene (β*-actin*) was used to estimate the average copy value of mtDNA/per cell. This technique allows the calculation of average mtDNA copy number/cell without the necessity to estimate cell number. Primers and probes were obtained from Sigma-Aldrich, Saint Louis, MA, USA on the base of sequences mouse genes used by Aiken et al.^31^ (Table 2). Probes were labelled fluorescently with carboxyfluorescein (FAM) (5′ end) and carboxytetramethylrhodamine (TAMRA) (3′ end). TaqMan Master Mix (Applied Biosystems, Foster City, CA, USA) was used for the reaction. The PCR conditions were following: initial steps at 50 °C for 2 min and 95 °C for 15 min, followed by 40 cycles of 15 s at 95 °C and 1 min at 65 °C. Standard curves were created separately for every sample from a dilution series to ensure the efficiency of reaction. The cycle threshold (CT) value for *β-actin* was subtracted from that for *Cytb* to give the value ΔCt. Average mtDNA copy number^30^ per nuclear genome (two actin gene copies) is calculated as 2 × 2^−(ΔCt)^.

**Table 2.** Primers and probes for mtDNA copy analysis.

Genes	Sequence (5′→3′)
*Cytochrome B*	F: TTTTATCTGCATCTGAGTTTAATCCTGT
	R: CCACCTCATCTTACCATTTATTATCGC
*Cytochrome B* probe	FAM-AGCAATCGTTCACCTCCTCTTCCTCCAC-TAMRA
β*-actin*	F: GGAAAAGAGCCTCAGGGCAT
	R: CTGCCTGACGGCCAGG
β*-actin probe*	FAM-CATCACTATTGGCAACGAGCGGTTCC-TAMRA

FAM: carboxyfluorescein; TAMRA: carboxytetramethylrhodamine.

### 2.6. Statistical development of the results

All analyses were performed with Statistica software (StatSoft10, Tulsa, OK, USA). Student’s *t*-test was used for comparison between groups. Pearson’s correlation test was applied to measure the relationship between investigated parameters. Values of *p* < .05 were considered significant. Data are presented as a mean ± SD, and triplicate repetition of the experiments was done.

PCR data were analysed by DataAssist Sft. (Ver. 3.01, Applied Biosystems, Foster City, CA, USA) using ΔCt method^32^. The results were expressed as the mean of target to control gene expression ratio [fold changes (FC)]. For the evaluation of the oregonin effect, ΔΔCt method^32^ was applied for the comparison between control and experimental groups. Genes with fold change cutoff of ≥ 2 and *p* values ≤ 0.05 compared with the control were considered to be upregulated, while with FC ≤ 0.5 and *p* ≤ 0.05 were considered to be downregulated. FC values between 0.5 and 2 indicated no significant change in gene expression.

### 2.7. Molecular modelling

The possible binding mode of oregonin to DNMT1 was predicted by molecular docking. The crystallographic structure of human DNMT1 in complex with S-adenosyl-l-homocysteine (SAH) coded by PDB 4WXX^33^ was retrieved from the Protein Data Bank (www.rcsb.org/pdb) and used as rigid receptor. As in our previous work^34^, molecular docking simulations were performed by FRED program from OpenEye (version 3.0.1)^35^^,^^36^. The receptor was prepared with the *make_receptor* utility by centring the binding site on the SAH ligand. The 3D structures of curcumin (reference control) and oregonin were downloaded from PubChem^37^. Ligands conformational analysis was carried out with OMEGA from OpenEye (version 2.5.1.4)^38^^,^^39^ by storing up to 600 conformers including the initial structure, sampling hydrogen positions for –OH groups and using all other parameters at their default values.

## 3. Results

### 3.1. Oregonin

The lyophilic dried oregonin isolated from *A. incana* bark EtOAc extract and used in all investigations contained 90 ± 0.8% of oregonin (2) (C_24_H_30_O_10_, UV *λ*max 281 nm. ES-: *m/z* 477 [M-H]^−^, *M* = 478.50 g/mol, 5 ± 0.1% of 1,7-bis-(3,4-dihydroxyphenyl)-3-hydroxyheptane-5-O-β-d-xylopyranoside (1) (C_24_H_32_O_10_, UV *λ*max 282 nm. ES-: *m/z* 479 [M-H]^−^, *M* = 480.52 g/mol) and 4 ± 0.1% of hirsutanolol (3) (C_19_H_22_O_6,_ UV λmax 281 nm. ES-: *m/z* 345 [M-H]^−^, *M* = 346.38 g/mol) (Figure 1).

The oregonin powder from *A. incana* showed high antioxidant activity. The following IC_50_ concentrations (required to scavenge 50% of free radicals) were reported: in ABTS^•+^ 3.14 ± 0.13 mg·mL^−1^ and in DPPH^•^ 4.52 ± 0.16 mg·mL^−1^ respectively compared to synthetic antioxidant trolox (a water-soluble vitamin E analogue), whose IC_50_ values in tests were 4.01 ± 0.12 (ABTS) mg·mL^−1^ and 4.72 ± 0.12 (DPPH) mg·mL^−1^.

### 3.2. Effect of oregonin on viability of cells *in vitro*

**Figure 3.**
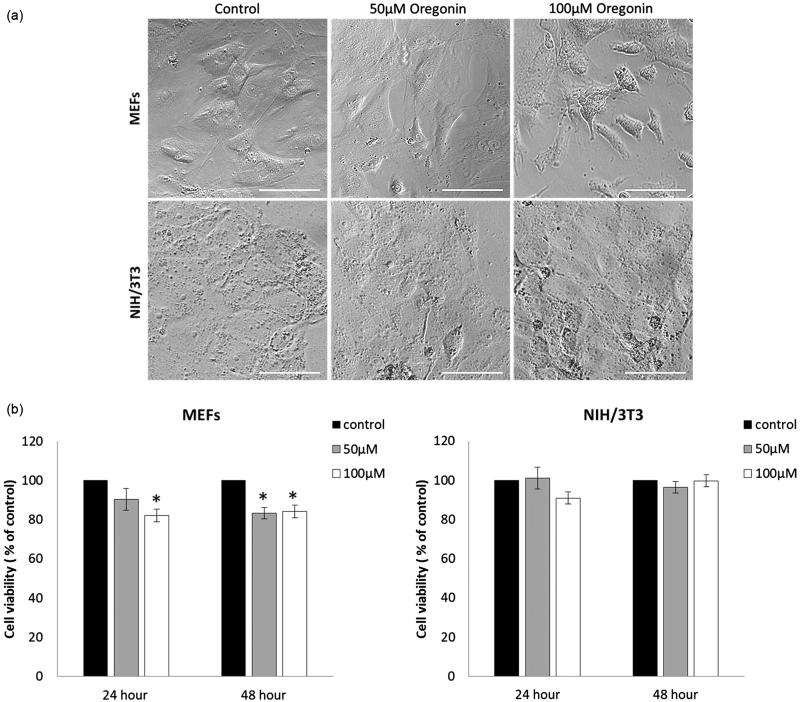
Effect of oregonin on MEFs and NIH/3T3 cells. (a) Cells grown 48 h in absence or presence of oregonin did not show significant changes in morphology. (b) Cell viability in MEFs and NIH/3T3 cells treated with 50 and 100 µM oregonin.

Untreated control MEFs exhibited normal fibroblast morphology at 48 h (Figure 3(a)). Upon incubation of MEFs with increasing concentrations of oregonin for 48 h, apoptotic morphological changes in a few cells were observed. The shapes of the oregonin treated NIH/3T3 were similar to those of the untreated cells. The potency of oregonin to reduce cell viability of MEFs and NIH/3T3 is shown in Figure 3(b). After 24 h treatment with 100 µM oregonin, MEFs viability was decreased by 18% (*t*-test *p* < .05). After 48 h of oregonin treatment in concentrations 50 and 100 µM, MEFs viability declined significantly by 16.7% and 15.8%, respectively. Both concentration of oregonin showed no growth inhibiting effect in NIH/3T3 cells after treatment of 24 and 48 h.

### 3.3. Effect of oregonin on the Dnmts expression and mtDNA copy number

**Figure 4.**
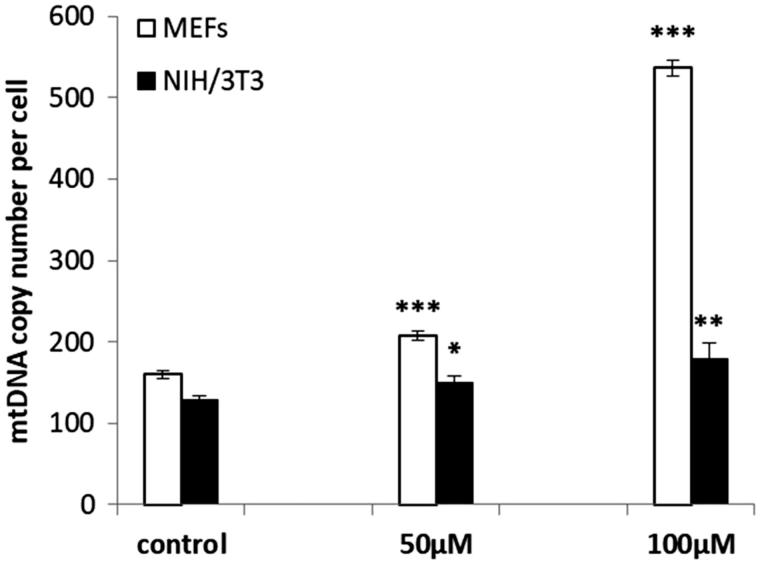
mtDNA copy numbers in MEF and NIH/3T3 cells treated with oregonin. The data are presented as a mean ± SD of three independent experiments. *p < .05; **p < .01; ***p < .001 compared to control.

A significant increase of mtDNA copy numbers in oregonin treated MEF and NIH/3T3 cells was found (Figure 4). The highest effect was observed in MEFs incubated with 100 μM oregonin (increase with 3.37 fold). The number of the mtDNA copy in NIH/3T3 cell at 50 μM and 100 μM increased only with 0.17 and 0.39 fold, respectively.

**Figure 5.**
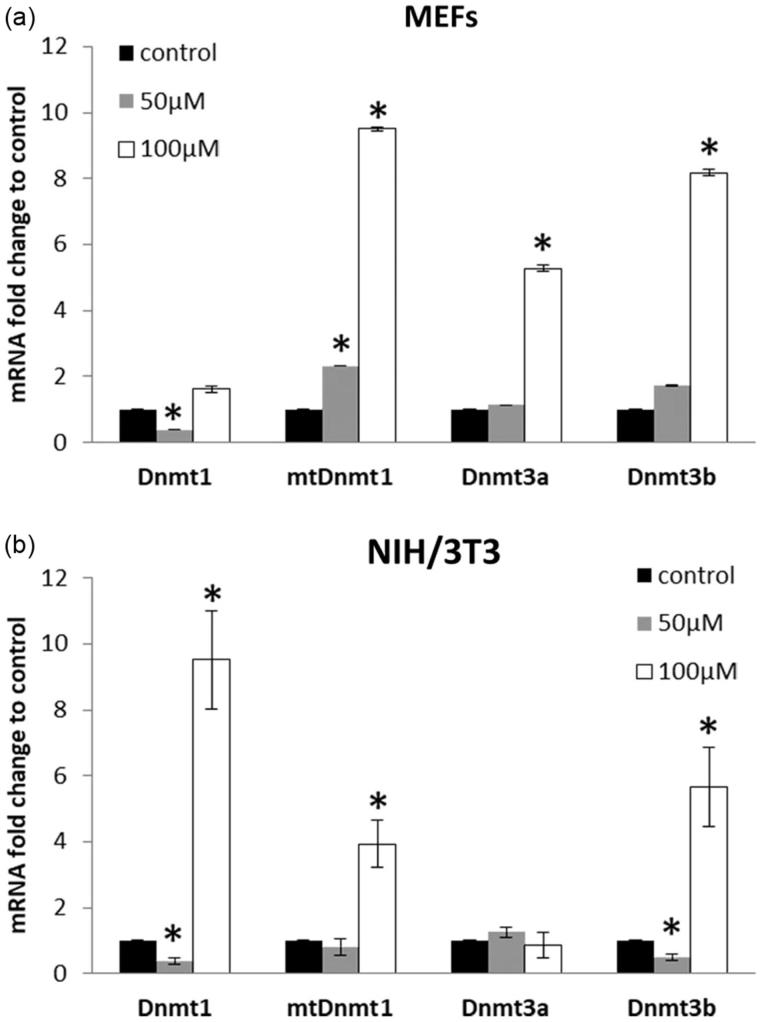
DNA methyltransferases mRNA expression in MEF (a) and NIH3T3 (b) cells incubated with oregonin in doses 50 and 100 μM for 24 h. The data are presented as a mean ± SD of three independent experiments. Fold change cut-off of ≥2 and *p* values ≤ 0.05 compared with the control were considered to be upregulated, while with FC ≤ 0.5 and *p* ≤ .05 were considered to be downregulated. *p < .05.

Purified oregonin in dose 50 μM down-regulated mRNA transcripts of Dnmt1 in both NIH/3T3 and MEFs and had no effect on the Dnmt3a at 24 h of incubation (Figure 5(a,b)).

The *mtDnmt1* transcripts in NIH/3T3 cells were unchanged (FC = 0.8) while in MEFs their level increased (FC = 2.31, *p* < .05). The *Dnmt3b* transcripts were unaffected in MEFs, but downregulated in NIH/3T3 cells. Oregonin in dose 100 μM up-regulated *mtDnmt1* and *Dnmt3b* in both cell types (FC > 2.0, *p* < .05). MEFs treated with this concentration of oregonin showed increased expression of *Dnmt3a*, but had no effect on *Dnmt1* mRNA transcripts. At the same experimental conditions (100 μM) in NIH3T3 cells, a drastic increase of *Dnmt1* (FC = 9.51, *p* < .05) was observed, whereas no alterations were found on *Dnmt3a* mRNA transcripts.

The correlative analysis showed the different relationships between expressed *Dnmts* in MEF and NIH/3T3 cells. A high positive correlation was determined between *mtDnmt1* and *Dnmt3b* as well as between *Dnmt3b* and *Dnmt3a* in MEFs (Table 3). In NIH/3T3 cells, we observed a similar relationship between *mtDnmt1* and *Dnmt3b*. Additionally, the changes in the expression of total Dnmt1 closely correlated with changes in the expression of *mtDnmt1* and *Dnmt3b*.

**Table 3.** Correlative relationship between expression of *Dnmts* in NIH/3T3 and MEF cells.

FC	*mtDnmt1*	*Dnmt3b*	*Dnmt1*	*Dnmt3a*
MEFs			*R*	
*mtDnmt1*	–	0.995	0.785	0.993
		***p* = 0.033**	*p* = 0.42	*p* = 0.074
*Dnmt3b*	0.995	–	0.816	0.997
	*p* = 0.033		*p* = 0.39	*p* = 0.041
*Dnmt1*	0.785	0.816	–	0.852
	*p* = 0.42	*p* = 0.39		*p* = 0.35
*Dnmt3a*	0.993	0.997	0.816	–
	*p* = 0.074	*p* = 0.041	*p* = 0.39	
NIH/3T3			*R*	
*mtDnmt1*	–	0.998	0.999	−0.803
		*p* = 0.02	***p* = 0.003**	*p* = 0.406
*Dnmt3b*	0.998	–	0.998	−0.821
	*p* = 0.02		*p* = 0.017	*p* = 0.387
*Dnmt1*	0.999	0.998	–	−0.806
	*p* = 0.003	*p* = 0.017		*p* = 0.403
*Dnmt3a*	−0.803	−0.821	−0.806	–
	*p* = 0.406	*p* = 0.387	*p* = 0.403	

*R*: Pearson’s correlation coefficient; FC: folder change; significance of results *p* < .05.

The interesting finding was the definition of the close relationship between mtDNA copy number and expression of *mtDnmt1* and *Dnmt3b* in MEFs (Table 4).

**Table 4.** Correlation between mtDNA copy number and *Dnmts* expression in mouse fibroblasts.

FC	*mtDnmt1*	*Dnmt3b*	*Dnmt1*	*Dnmt3a*
MEFs		*R*		
Cn-mtDNA	0.999	0.999	0.797	0.995
	*p* = 0.012	*p* = 0.021	*p* = 0.413	*p* = 0.062
**NIH/3T3**	0.836	0.818	0.833	−0.344
Cn-mtDNA	*p* = 0.370	*p* = 0.389	*p* = 0.373	*p* = 0.776

*R*: Pearson’s correlation coefficient; Cn-mtDNA: copy number of mitochondrial DNA; FC: folder change; significance of results *p* < .05.

### 3.4. Molecular docking of oregonin to DNMT1

**Figure 6.**
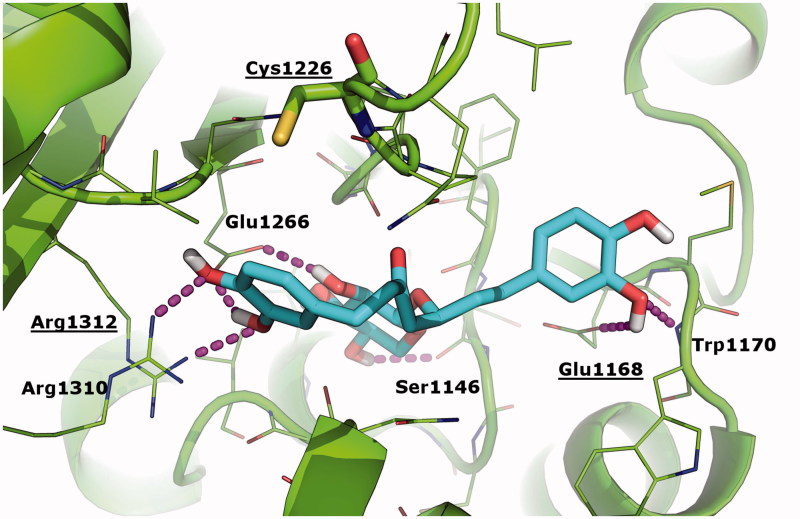
Binding mode of oregonin to DNMT1 crystallographic structure as predicted by molecular docking simulations. Oregonin is shown as cyan sticks; the crystallographic structure of DNMT1 (PDB: 4 WXX)^31^ is shown as green cartoon; residues within 5 Å from Oregonin are showed as green lines; Cys1226 is shown as green sticks. H-bonds are highlighted as magenta dashed lines; DNMT1 residues H-bonded to oregonin are labelled. Residues that are contacted by curcumin and its analogues in Liu et al.’s study^12^ are labelled and underlined.

The work of Liu et al.^12^ has clearly substantiated the possible covalent binding of curcumin to DNMT1 in correspondence of Cys1226 as a mechanism of hypomethylation. Based on the chemical similarity between curcumin and oregonin, and sustained by biological activity data described above, here we aimed to elucidate the possible binding of oregonin to DNMT1 and the respective pharmacophores. To this aim, we performed molecular docking simulations of curcumin (used as reference control) and oregonin towards the crystallographic structure of human DNMT1 in complex with SAH^33^. Results of our study were nicely in agreement with the binding mode of curcumin predicted by Liu et al.^12^ towards the DNMT1 homology model (data not shown), and suggested for the first time the geometric fitting of oregonin within the catalytic site of DNTM1 (Figure 6). In particular, oregonin binds in proximity of the cofactor binding cleft of DNMT1 and partially overlaps with the crystallographic binding conformation of SAH. This interaction is reinforced by a number of H-bonds established with residues Ser1146, Glu1168, Trp1170, Glu1266, Arg1310, and Arg1312 (Figure 6). The sugar moiety occupies a sub-pocket of DNMT1 and nicely overlaps with the homocysteine moiety of SAH, thus further suggesting that DNMT1 inhibition by oregonin may be due to the competition with the cofactor SAH, as already observed^34^ for different type of epigenetics regulators. Similar to curcumin, oregonin does not interact directly with the key residue Cys1226, even though it is located in close proximity of oregonin in docking results (minimum distance between the thiol group of Cys1226 and oregonin is 3.7 Å). Moreover, oregonin establishes H-bonds with Glu1168 and Arg1312, which have been already highlighted as crucial anchor points for the binding of curcumin derivatives in the work of Liu et al.^12^ Different from curcumin, oregonin does not possess chemically reactive groups that might interact covalently with Cys1226, thus the binding of oregonin to DNMT1 is expected to be non-covalent and reversible. Finally, the Chemgauss4 affinity score calculated by the docking program is slightly better for curcumin than oregonin (−11.25 vs. −10.99, respectively; the lower the better).

## 4. Discussion

### 4.1. Cell viability

The results of the cell viability test are in agreement with data obtained for the oregonin extracted from others genus of *Alnus*. The period of incubation 72 h and the concentrations of oregonin from *Alnus japonica* between 0 and 50 µM had no negative effects on the viability and morphology of endothelial cells^40^. Under the *in vivo* studies have shown the low toxicity of oregonin from *A. glutinosa*^41^ in the experiments with brine shrimps and from *A. japonica*^7^ – with specific pathogen-free ICR mice. It was reported^42^ that diarylheptanoids from *Alnus nepalensis* show high biological activities without any side effect at high oral dose. Compared to other polyphenol with similar structure, particularly curcumin, oregonin is less toxic. In fact, curcumin in dose 50 μM in *in vitro* experiments^43^ reduced the cell viability more than 50%, whereas in our study the lowest percent of viable cells treated with oregonin in dose 50 μM was more than 80%. The results from our study clearly showed that oregonin from bark of *A. incana* in doses of 50 and 100 μM had no effect on the viability of NIH3T3 cells in the frame of 48 h and expressed middle toxically effect on MEFs (up to 18%).

### 4.2. mtDNA copy number and *Dnmts*

Changes in mtDNA copy number have been reported in a broad range of human diseases^44^, such as diabetes and its complications, obesity, cancer, HIV complications and ageing. Our interest in the investigation of this parameter was triggered by the evidence of important role of mitochondria in the DNA methylation process and remarkable antioxidant properties of oregonin. Results described in this work clearly show the ability of oregonin to increase the mtDNA content in the investigated cells although this effect is quantitatively dependent on the cell type, as NIH/3T3 cells showed more genomic stability than MEFs. The drastic increase of mtDNA content in MEFs treated with 100 μM was accompanied by a decrease of cell viability at 24 h. Most likely, there is an adaptive response to increase oxidative stress by an enhancement of mitochondrial biogenesis due to toxic dose of oregonin^44^. On the other hand, these changes also correspond to the increase of Dnmts expression in MEFs. Our study highlighted for the first time the close relationship between changes in mtDNA content and expression of Dnmts mRNAs, which could be explained by considering that DNMTs enzymatic function is depended on the methyl donor SAM, whose proper production is guaranteed also by mitochondria^18^. The dependence of the DNA methylation process on the mitochondria content is confirmed by Smiraglia et al.^19^, who analyse the methylation status of several genes in response to the depletion and repletion of mtDNA. However, the mechanism provoked by oregonin to increase the mtDNA replication is still unclear. The replication of the mitochondrial genome is regulated via the actions of a combination of gene expression coding in mtDNA and nuclear DNA such as d-loop region of mtDNA, TFAM, RNA polymerase (POLRMT), DNA polymerase POLG, transcription factors 1 and 2 (TFB1Mand TFB2M), nuclear respiratory factors 1 and 2 (NRF-1, NRF-2) and peroxisome proliferator-activated receptor gamma coactivator 1-alpha (PGC1-α)^45–48^. Oregonin could affect these genes via genetic or epigenetic pathways due to its ability to change the expression of *Dnmts*. Additionally, the anti-oxidative properties of oregonin result in an alteration of the mRNA expression of several antioxidant-related genes and oxidative stress responsive genes^10^, which could explain the increase in mtDNA copy number through the communication between nuclear and mitochondrial genes.

Changes in the mtDNA content and expression of Dnmts mRNA transcripts may be also related to mtDNA methylation. There are very few studies that describe the role of DNMTs in the regulation of the mtDNA methylation, and their results are often questionable by the lack of clear understanding of the regulation mechanism. However, the localisation of DNMTs to mitochondria in different cells has been reported. The mtDNMT1 was defined in the mitochondria of MEF and human colon carcinoma cells^49^, the DNMT3a localises to mitochondria in mouse brain and spinal cord^50^ and to mitochondria in muscle tissue of human^51^. Our results also show that in MEF cells there is the closest correlation between changes of the mtDNA content and expression of *mtDnmt1* mRNA transcripts.

The novelty of our study is the analysis of the oregonin effect on the expression of the mRNA transcripts of mitochondrial isomeric form of DNA methyltransferase1-*mtDnmt1*. Both oregonin concentrations up-regulated the level of the mtDnmt1 transcripts in MEFs. In NIH/3T3 cells, the increased level of mitochondrial isoform mRNA was provoked only by 100 μM of oregonin. Recently accumulated data^21^^,^^52^ reveal that external factors can alter mtDNA methylation followed by changing the mitochondrial genes expression. This process is mediated by mtDnmt1. In accordance with data of Shock et al.^49^, hyper expression of *mtDnmt1* in mouse fibroblasts related to the alteration in the expression of *Nd1* and *Nd6* genes, coding in mitochondria. Additionally, authors underlined that *mtDnmt1* is sensitive to regulation by activators that respond to oxidative stress. The well-known anti-oxidative properties of oregonin^1^^,^^10^ may further explain the overexpression of the *mtDnmt1* transcripts, as observed in our work.

Here, we showed that polyphenol oregonin affected the expression of DNMTs transcripts in both NIH/3T3 cells and MEFs but in some different ways. Its effect is depended on concentration and specific of cells genotype. The drastic increase of the DNA methyltransferase mRNA expression (FC between 4.9 and 9.5) in both types of cells treated with 100 μM of oregonin allows proposing that the molecule exerts some epigenetic toxicity. The increased level of DNMTs could lead to DNA hypermethylation, which occurrence at promoter CpG islands is a major epigenetic mechanism in silencing the genes expression^53^^,^^54^. That could have both side effects in dependence of silenced genes. The silence of the viable important genes could disturb the cells and provoke the diseases development. However, suppression of “negative” genes could have therapeutic effects.

The main data^13^^,^^56^^,^^57^ about the influence of polyphenols, including curcumin, on the DNA methylation stressed a hypomethylation effect due to downregulation of the *Dnmt1* mRNA transcripts, which directly correlated with decrease of the DNMT1 protein expression. Here, we observed similar effects for *Dnmt1* mRNA in two different cell lines treated with oregonin at 50 μM. *In silico* study^12^ of the interaction between curcumin and DNMT1 suggested that curcumin covalently blocks the catalytic thiolate of DNMT1 to exert its inhibitory effect on DNA methylation. In line with Zheng et al.^55^ results, reported the suppression of DNA methyltransferase 3 b by curcumin, we also defined the decrease of *Dnmt3b* mRNA transcripts (FC = 0.5, *p* < .05) in NH/3T3 cells treated with 50 μM of oregonin. In analogy to Liu et al.^12^, here we used molecular modeling to predict the possible binding of oregonin to DNMT1. Although oregonin does not possess chemically reactive groups as curcumin, docking results clearly showed that the two compounds might bind within the catalytic site of DNMT1 in a similar conformation, and partially overlapping with the binding of the cofactor SAH/SAM. Based on the chemical similarity between curcumin and oregonin, here we pinpointed the pharmacophores that may be relevant for binding to DNMT1 and inhibiting its catalytic function to provide hypomethylation effects.

## 5. Conclusion

The new properties of the purified oregonin, extracted from the bark of *A. incana*, were confirmed in this study. The oregonin altered the epigenetic marks related to the DNA methylation, especially, expression of DNMTs mRNAs and mtDNA content in MEFs in dependence on used concentration and cell genotype. Molecular docking predicted the ability of oregonin to bind within the catalytic active site of DNMT1, which suggest that the compound may inhibit DNMT1 enzymatic activity.

However, the development of oregonin as a substance with potential to regulate the epigenetic changes, needs further investigations, particularly to elucidate the pathways of other epigenetic mechanisms (histone acetylation, chromatin remodelling) and genes involved in these processes. Additionally, research is needed to determine the appropriate dose for treatment without cytotoxic effect.
